# Community engagement to co-design and deliver public health interventions in newly arrived migrants

**DOI:** 10.1093/eurpub/ckac129.116

**Published:** 2022-10-25

**Authors:** A Crawshaw

**Affiliations:** The Migrant Health Research Group, St Georges, University of London, London, UK

## Abstract

Alison Crawshaw will talk about her participatory research that uses design thinking to develop community-based strategies to improve health outcomes in migrant populations. She will summarise the benefits of using community-based approaches and co-designing public health initiatives with affected migrant communities, bringing in lessons learned from a recent project with the Congolese community in London.

